# Efficacy of Yupingfeng powder in the treatment of bronchial asthma in adults: a systematic review and meta‐analysis

**DOI:** 10.3389/fphar.2026.1757474

**Published:** 2026-05-01

**Authors:** Qianqian Zhang, Nianzhi Zhang, Ying Zheng, Jing Zhou, Ling Liu, Dong Gang

**Affiliations:** 1 First Clinical Medical College of Anhui University of Chinese Medicine, Hefei, China; 2 Department of Respiratory and Critical Care Medicine, The First Affiliated Hospital of Anhui University of Chinese Medicine, Hefei, China

**Keywords:** bronchial asthma, conventional biomedical therapy, meta-analysis, systematic review, Yupingfeng powder

## Abstract

**Background:**

Yupingfeng Powder (YPF), a classical Chinese herbal formula composed of Astragalus membranaceus, Atractylodes macrocephala, and Saposhnikovia divaricata, has demonstrated potential efficacy in the treatment of adult bronchial asthma when combined with conventional biomedical therapy (CBT), as indicated by multiple recent clinical studies. However, existing research is limited by small sample sizes, inconsistent methodological quality, and non-uniform syndrome differentiation, resulting in insufficient evidence strength. Moreover, traditional meta-analysis methods struggle to address study heterogeneity, and its definitive efficacy remains inadequately validated.

**Objective:**

To systematically evaluate the clinical efficacy and safety of YPF combined with CBT in the treatment of adult bronchial asthma, and to clarify the heterogeneity sources.

**Methods:**

Strictly adhering to the PRISMA 2020 guidelines, the study protocol was registered in PROSPERO (registration number: CRD420251172600). A systematic search was conducted in seven databases (PubMed, Embase, Cochrane Library, China, etc., up to 20 October 2025), and randomized controlled trials (RCTs) comparing YPF+CBT with CBT alone in adults with bronchial asthma were included. The Cochrane ROB 2.0 tool was used to assess the risk of bias, and meta-analysis was performed using RevMan 5.4 and Stata 18 software. Subgroup analysis and Meta regression were employed to explore the sources of heterogeneity.

**Results:**

A total of 20 RCTs were ultimately included, involving 1,896 participants (950 in the intervention group and 946 in the control group). The meta-analysis revealed that the YPF+CBT group demonstrated significantly higher clinical overall response rate compared to the CBT group [risk ratio (RR) = 1.20, 95% CI (1.16, 1.25), P < 0.001, I^2^ = 0.0%]. Significant improvements were observed in pulmonary function parameters (FEV1, FVC, FEV1/FVC, PEF) and asthma control test (ACT) scores [SMD were 1.32, 1.78, 1.09, 1.15, and 1.80, respectively, all P < 0.05]. No statistically significant difference was found in the CD4+/CD8+T lymphocyte subset ratio between the two groups [SMD = 0.50, 95%CI(-0.44, 1.44), P = 0.301]. Subgroup analysis and meta-regression confirmed that treatment duration (P = 0.03) and study year (P = 0.043) were the primary sources of heterogeneity. In terms of safety, only two studies reported mild adverse events (e.g., gastrointestinal discomfort, dry mouth), with no significant difference in incidence between the groups and no serious adverse events. Clinical significance analysis showed that the clinical overall response rate in the YPF+CBT group was 86.8%, which was compared to 72.3% in the CBT group, with an absolute risk reduction (ARR) of 14.5%, indicating clear clinical benefits of this combination therapy.

**Conclusion:**

YPF combined with CBT can significantly improve clinical efficacy, pulmonary function, and symptom control in adult bronchial asthma patients. However, due to limitations such as moderate methodological quality of the included studies, geographical constraints, and insufficient adverse event data, the conclusions require further validation through high-quality RCTs with large sample sizes, double-blind design, and placebo control.

**Systematic Review Registration:**

https://www.crd.york.ac.uk/prospero/display_record.php?RecordID=1172600, identifier CRD420251172600.

## 1 Introduction

Bronchial asthma is a highly prevalent respiratory disease worldwide, characterized by chronic airway inflammation, airway hyperresponsiveness, reversible airflow limitation, and airway remodeling, driven by both genetic and environmental factors (Jayasooriya et al., 2025). Its symptoms exhibit significant variability with the degree of airflow limitation. In 2021, the global confirmed cases reached 270 million, with 450,000 deaths (Yuan L. et al., 2025). In China, the prevalence of asthma among adults aged 20 and above is 4.2%, with a total of 45.7 million cases, but less than 30% achieve good control. The medical burden associated with acute exacerbations has become a major public health challenge (Huang et al., 2019). The pathogenesis of asthma is complex, involving multiple factors such as immune imbalance. Poor long-term control leads to progressive decline in lung function, increasing the risk of acute exacerbations and death (Eger et al., 2022).

Current standardized asthma treatment follows the GINA guidelines (Venkatesan, 2025), with inhaled corticosteroids (ICS) combined with long-acting beta2-agonists (LABA) as the core control medications. However, clinical challenges remain: long-term ICS use may lead to adverse effects such as osteoporosis (Rogliani et al., 2022), 10%–15% of patients have “refractory asthma” (Peters et al., 2007), overuse of SABA is associated with disease exacerbation (Krings and Beasley, 2024), and some patients exhibit poor medication adherence. Therefore, identifying adjunctive treatment options that reduce dependence on Western medications has become a key focus in clinical research.

Yupingfeng Powder (YPF), a classical Chinese herbal formula composed of Astragalus membranaceus, Atractylodes macrocephala, and Saposhnikovia divaricata, was first documented in the “Danxi’s Heart Method” and is known for its effects of replenishing qi, consolidating the exterior, and stopping sweating (Pengfei et al., 2024). Recent clinical studies have suggested that its combination with conventional biomedical therapy (CBT) can improve asthma clinical control rates and enhance pulmonary function (Minjie et al., 2020; Yang et al., 2021; Li et al., 2022). However, existing research is limited by small sample sizes, inconsistent methodological quality, and non-uniform syndrome differentiation, resulting in insufficient evidence strength (Chao et al., 2013). Traditional meta-analyses struggle to address the heterogeneity in TCM botanical formulations research, leading to its exclusion from authoritative guidelines (Wu et al., 2020). As the first systematic review and meta-analysis targeting YPF for adult asthma treatment, this study systematically evaluates its efficacy and safety through subgroup analysis, meta-regression, and sensitivity analysis, clarifies the sources of heterogeneity, and provides evidence-based insights for optimizing integrated TCM-Western medicine treatment regimens and updating guidelines.

## 2 Materials and methods

### 2.1 Classification verification of key plant medicinal materials

The classification verification of key plant medicinal materials in YPF has been completed through the Medicinal Plant Name Service (MPNS, https://mpns.science.kew.org/mpns-portal/) and the MYCOBANK database (https://www.mycobank.org/). Specific details are provided in Table 1.

**TABLE 1:** Classification and Characteristic Information of Core Botanical components in YPF.

Chinese botanical drugs name	Full botanical drug species name	Part of botanical drugs	Main markers or active metabolites	Efficacy	Taxonomic validation method	Authoritative database validation ID
Huang Qi	*Astragalus membranaceus* (Fisch.) Bunge [Fabaceae; Astragali Radix]	Root	Astragaloside IV (Tan et al., 2020), Astragalus polysaccharides (Wei et al., 2023), Calycosin-7-glucoside (Chen D. et al., 2025)	Immunomodulation (Li et al., 2020), anti-inflammatory (Adesso et al., 2018), anti-oxidation (Li et al., 2019), enhancing pulmonary function (Shi et al., 2024)	MPNS + MYCOBANK database cross-verification	MPNS: 10000245; MYCOBANK: MB839412
Bai Zhu	*Atractylodes macrocephala* Koidz. [Asteraceae; Atractylodis Macrocephalae Rhizoma]	Rhizome	Atractylenolide III (Zhang et al., 2025), Atractylodes polysaccharides (Kong et al., 2025), β-eudesmol (Ke et al., 2025)	Anti-inflammatory (Yuan et al., 2024), regulating intestinal flora (Feng et al., 2020), anti-tumor (Guo et al., 2020)	MPNS + MYCOBANK database cross-verification	MPNS: 10000328; MYCOBANK: MB840157
Fang Feng	*Saposhnikovia divaricata* (Turcz.)Schischk. [Apiaceae; Saposhnikoviae Radix]	Root	Prim-O-glucosylcimifugin (Prim-O-glucosylcimifugin attenuates intestinal fibrosis by modulating TGF-β/MAPK signaling and ECM remodeling, 2025), 4′-O-β-D-glucosyl-5-O-methylvisamminol (Yoo and Keum, 2019), Volatile oils (Limonene, α-pinene) (Wang S-Y et al., 2025)	Anti-allergic (Li et al., 2022), anti-oxidation (Zhang et al., 2008), analgesic (Oh et al., 2021)	MPNS + MYCOBANK database cross-verification	MPNS: 10000412; MYCOBANK: MB841029

### 2.2 Compliance with ConPhYMP guidelines

As direct morphological identification of raw medicinal materials is not feasible in a systematic review (we do not have access to original plant samples), we validated the taxonomic consistency of botanical names reported in included studies against these databases—confirming full scientific names (including authorities), family classifications, and pharmacopeial designations to ensure taxonomic accuracy (Supplementary Table S2). This approach adheres to ConPhYMP Guideline Item 2 (taxonomic authentication) for systematic reviews, where reliance on database validation and original study reporting is the only viable method.

### 2.3 Literature retrieval strategy

To comprehensively retrieve relevant literature, we systematically searched seven databases: PubMed, Embase, Web of Science, Cochrane Library, Sinomed, China National Knowledge Infrastructure (CNKI), and Wanfang Data Knowledge Service Platform, with the search period covering the establishment of each database to 20 October 2025. The retrieval strategy combined subject terms with free words, focusing on core concepts such as “YPF” and its related dosage forms, as well as “bronchial asthma.” Additionally, we manually searched the reference lists of included literature and also searched gray literature databases such as the China National Knowledge Infrastructure Master/Doctoral Dissertation Database and the China Clinical Trial Registry (ChiCTR). No relevant unpublished randomized controlled trials were found. For details of the specific retrieval strategy, see Supplementary Table S3.

### 2.4 Inclusion and exclusion

Criteria In this study, all preparations related to YPF were formulated with Astragalus membranaceus, Atractylodes macrocephala, and Saposhnikovia divaricata as core components, with dosage forms including granules, decoctions, and dripping pills. Modified decoctions were adjusted only with minor additions of adjuvant botanical ingredients without altering the core pharmacological compatibility. The PICO principle was adopted to clarify the inclusion and exclusion criteria.

#### 2.4.1 Inclusion criteria

Research type (P): RCTs, with language limited to Chinese or English.

Study subjects (I): Adult patients aged ≥18 years with bronchial asthma diagnosed according to the Global Initiative for Asthma (GINA) guidelines (Venkatesan, 2025), regardless of gender, disease duration, or severity; predominantly with TCM syndrome of lung-qi deficiency or lung-spleen qi deficiency, both meeting the indications of YPF for “impaired defensive qi and lung qi deficiency”; all patients had complete baseline data, and the baseline characteristics (age, gender, disease duration, etc.) of the two groups were comparable.

Intervention Measures (I): The experimental group received YPF or its common dosage forms (granules, decoctions, drop pills, capsules, etc.) as monotherapy or in combination with CBT. The dosage and duration of YPF were not restricted, but must be clearly reported.

Control measures (C): The control group received CBT (standardized treatment in accordance with GINA guidelines, including inhaled corticosteroids/long-actingβ2-agonist combination, leukotriene receptor antagonists, or monotherapy with inhaled corticosteroids) or placebo, with intervention methods and duration consistent with those in the trial group. The specific protocol must be clearly documented in the study protocol.

Outcome Measures (O):The primary outcome measure was the overall clinical response rate (defined according to the “Guidelines for the Prevention and Treatment of Bronchial Asthma (2024 Edition)” (Yan and Zhang, 2025), categorized as clinically controlled, significantly effective, effective, or ineffective, calculated as (number of clinically controlled+significantly effective+effective) cases/total number of cases×100%). The secondary outcome measures included pulmonary function parameters (forced expiratory volume in 1 s [FEV1], forced vital capacity [FVC], FEV1/FVC ratio, and peak expiratory flow [PEF]). The secondary outcome measures also included T lymphocyte subsets (CD4^+^, CD8+, CD4+/CD8+ratio), Asthma Control Test (ACT) scores, and traditional Chinese medicine (TCM) syndrome scores.

#### 2.4.2 Exclusion criteria

Non-randomized controlled trials (e.g., reviews, case reports, animal experiments, cohort studies, cross-sectional studies, etc.); unclear diagnostic criteria for study subjects, or coexisting with other severe respiratory diseases, cardiovascular and cerebrovascular diseases, hepatic or renal insufficiency, immunodeficiency diseases, etc.; intervention in the experimental group involving modified YPF (with added or reduced core botanical materials), or combined with other TCM botanical formulations compound and external treatment methods; incomplete data, inability to obtain full-text articles, or duplicate publications.

### 2.5 Data extraction and quality assessment

After standardized training, two researchers (Q. Q. Zhang and Y. Zheng) independently used a pre-designed standardized data extraction form to collect information, including study baseline characteristics, subject characteristics, intervention and control measures, outcome indicators, and bias risk-related data. Cross-verification was conducted after data extraction, with discrepancies resolved through consultation or arbitration by a third researcher (J. Zhou). For missing or questionable data, the corresponding author of the original literature was contacted via email for supplementation. Data that remained unresponsive or inaccessible after three attempts was treated as missing. A database was established using Excel 2019, with double-entry verification to ensure an error rate controlled within 5%.

Using the bias risk assessment tool (ROB 2.0) recommended by the Cochrane Collaboration, the two aforementioned researchers independently conducted methodological quality assessments of the included RCTs.The evaluation areas included randomization processes, deviations from protocol, missing outcome data, outcome measures, and selective reporting of results, which were categorized as low risk, “high risk,” “or some concern,” respectively. Disagreements were resolved through discussion.

### 2.6 Statistical analysis

Statistical analysis was performed using Review Manager (RevMan) 5.4 and Stata 18 software, with an alpha level of 0.05. Dichotomous variables were described using the risk ratio (RR) and 95% confidence interval (95% CI), while continuous variables were presented as standardized mean difference (SMD) and 95% CI based on the consistency of measurement tools and units. The Q-test combined with the I^2^ statistic was employed to assess heterogeneity, with fixed-effect models used when I^2^ ≤ 50% and random-effects models when I^2^>50%. Subgroup analysis and meta-regression were conducted to explore the sources of heterogeneity. Sensitivity analysis was performed by sequentially excluding individual studies and swapping effect models to verify the robustness of the results. Publication bias was assessed using funnel plots and Egger’s linear regression test.

## 3 Results

### 3.1 Literature screening process and preliminary screening

**FIGURE 1:**
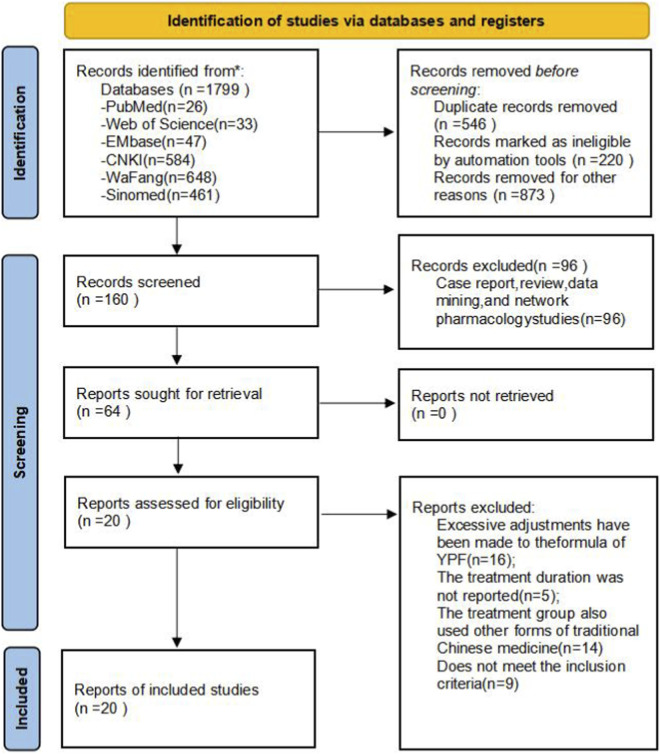
Screening flowchart.

A total of 1,799 relevant articles were obtained through the literature screening process. After excluding duplicate articles, 1,253 articles remained. By reviewing titles and abstracts, 1,093 obviously irrelevant articles were excluded, leaving 160 articles for full-text review. Further screening eliminated 140 articles that did not meet the RCT criteria, had inappropriate intervention measures, or incomplete data. Ultimately, 20 studies met the inclusion criteria and were selected for this systematic review and meta-analysis. The literature screening process is detailed in Figure 1.

### 3.2 Study characteristics

Twenty studies (Zheng and Zhang, 2015; Zhong et al., 2016; Zhang, 2018; Wang et al., 2019; Zhang et al., 2019; Zhou et al., 2019; Minjie et al., 2020; Guo, 2021; Xin and Wenjuan, 2021; Yang et al., 2021; Fang and Dong, 2022; Li et al., 2022; Ma, 2022; Song et al., 2022; Jun and Yu, 2022; Mulan et al., 2023; Fu and Dong, 2024; Wang and Wang, 2024; Chen, 2025) were published between 2015 and 2025, involving a total of 1,896 participants (950 in the intervention group and 946 in the control group), with treatment durations ranging from 2 weeks to 6 months. Among these, five studies focused on the acute exacerbation phase of bronchial asthma (Guo, 2021; Fang and Dong, 2022; Ma, 2022; Song et al., 2022; Fu and Dong, 2024), seven on the chronic persistent phase (Zheng and Zhang, 2015; Zhang, 2018; Wang et al., 2019; Zhou et al., 2019; Xin and Wenjuan, 2021; Jun and Yu, 2022; Xiuhua et al., 2025), and five on the clinical remission phase (Zhong et al., 2016; Minjie et al., 2020; Li et al., 2022; Mulan et al., 2023; Wang and Wang, 2024). Three studies did not specify the disease stage (Zhang et al., 2019; Yang et al., 2021; Chen, 2025). All experimental groups received YPF, Yupingfeng Granules, or Yupingfeng Dropping Pills combined with CBT, while the control group received CBT. In terms of dosage forms, 12 studies used Yupingfeng Granules (Zhong et al., 2016; Zhang et al., 2019; Zhou et al., 2019; Guo, 2021; Xin and Wenjuan, 2021; Fang and Dong, 2022; Ma, 2022; Song et al., 2022; Jun and Yu, 2022; Mulan et al., 2023; Fu and Dong, 2024; Wang and Wang, 2024; Chen, 2025), six used decoctions (Zheng and Zhang, 2015; Minjie et al., 2020; Guo, 2021; Yang et al., 2021; Li et al., 2022; Xiuhua et al., 2025) (including three modified YPF formulations (Zheng and Zhang, 2015; Minjie et al., 2020; Guo, 2021)), and two used dropping pills (Zhang, 2018; Wang et al., 2019). Regarding Western medical treatments, 20 studies employed seven asthma medications and one specific immunotherapy regimen (4 studies) (Zhong et al., 2016; Minjie et al., 2020; Wang and Wang, 2024; Chen, 2025), Budesonide/Fomoterol (5 studies) (Zhang, 2018; Fang and Dong, 2022; Ma, 2022; Jun and Yu, 2022; Fu and Dong, 2024), Salmeterol/Fluticasone (3 studies) (Zheng and Zhang, 2015; Yang et al., 2021; Mulan et al., 2023), Budesonide (2 studies) (Li et al., 2022; Song et al., 2022), Fluticasone (2 studies) (Wang et al., 2019; Zhang et al., 2019), and specific immunotherapy (1 study) (Xiuhua et al., 2025). Regarding outcome measures, 15 studies reported overall clinical efficacy rates (Zheng and Zhang, 2015; Zhong et al., 2016; Zhang, 2018; Wang et al., 2019; Zhang et al., 2019; Minjie et al., 2020; Xin and Wenjuan, 2021; Song et al., 2022; Jun and Yu, 2022; Mulan et al., 2023; Fu and Dong, 2024; Wang and Wang, 2024; Chen, 2025; Xiuhua et al., 2025), 14 reported FEV1 (Zhong et al., 2016; Zhang, 2018; Wang et al., 2019; Guo, 2021; Xin and Wenjuan, 2021; Fang and Dong, 2022; Li et al., 2022; Ma, 2022; Song et al., 2022; Mulan et al., 2023; Fu and Dong, 2024; Wang and Wang, 2024; Chen, 2025; Xiuhua et al., 2025), 7 reported FVC (Guo, 2021; Fang and Dong, 2022; Ma, 2022; Song et al., 2022; Mulan et al., 2023; Wang and Wang, 2024; Chen, 2025), 4 reported FEV1/FVC (Wang et al., 2019; Xin and Wenjuan, 2021; Fang and Dong, 2022; Song et al., 2022), 7 reported PEF (Zhang, 2018; Xin and Wenjuan, 2021; Li et al., 2022; Mulan et al., 2023; Fu and Dong, 2024; Wang and Wang, 2024; Xiuhua et al., 2025), 4 reported CD4+/CD8+ratio (Zhang et al., 2019; Minjie et al., 2020; Yang et al., 2021; Fang and Dong, 2022), and 4 reported ACT scores (Zhou et al., 2019; Li et al., 2022; Fu and Dong, 2024; Chen, 2025).

All included studies demonstrate consistent YPF composition and complete reporting of essential information, supporting the validity of pooling results: ① Essential component consistency: All 20 studies retain YPF’s three core components (Astragalus membranaceus, Atractylodes macrocephala, Saposhnikovia divaricata), the fundamental drivers of its therapeutic effect for asthma. Among these, 17 studies use unmodified YPF, while 3 modified studies only add mild adjuvants without removing or replacing any core botanical drug.② Complete composition reporting: Every original study explicitly lists the three core botanical materials and their daily dosages; for modified formulations, adjuvant type and dosage are also clearly documented.③ Therapeutic comparability guarantee: YPF’s efficacy depends on the synergistic effect of its three core components. Minor dosage variations of core botanical materials (within clinically accepted ranges for adult asthma) and mild adjuvants do not alter its core therapeutic direction (reducing inflammation, regulating immunity), which aligns with established TCM botanical formulations clinical practice norms. Detailed information on the included studies is provided in Table 2, and characteristics related to YPF preparations are listed in Table 3.

**TABLE 2:** Basic characteristics of the included studies.

Study	Year	Samplesize (T/C)		Age (T/C)		Therapy duration	Intervention measures (T/C)		Outcome measures	Asthma stage	Study design
Chen QL	2025	38	38	44.13 ± 5.69	43.92 ± 6.62	2 W	Yupingfeng granules+montelukast sodium	Montelukast sodium	①②③⑨	Not mentioned	RCT
Feng XY	2022	60	60	35.71 ± 2.44	36.17 ± 2.36	2 W	Yupingfeng granules+budesonide and formoterol fumarate powder for inhalation	Budesonide and formoterol fumarate powder for inhalation	①②③④⑥⑦⑧	Acute phase	RCT
Feng XH	2025	30	30	43.2 ± 9.8	42.5 ± 10.3	12 W	Yupingfeng powder+specific immunotherapy	Specific immunotherapy	①②	Chronic persistent phase	RCT
Fu ZL	2024	46	46	50.11 ± 6.45	49.36 ± 6.33	2 W	Yupingfeng granules+budesonide and formoterol fumarate powder for inhalation	Budesonide and formoterol fumarate powder for inhalation	①②⑨	Acute phase	RCT
Hou X	2021	30	30	52.23 ± 11.61	54.35 ± 13.36	8 W	Yupingfeng granules+budesonide suspension for inhalation	Budesonide suspension for inhalation	①②④⑤	Chronic persistent phase	RCT
Huang ML	2023	30	30	71.03 ± 3.41	70.83 ± 3.48	24 W	Yupingfeng granules+salmeterol Xinafoate and fluticasone propionate aerosol	Salmeterol Xinafoate and fluticasone propionate aerosol	①②③⑤	Remission period	RCT
Li MJ	2020	40	40	14.90 ± 2.04	25.36 ± 4.20	12 W	Modified Yupingfeng Powder+Montelukast sodium	Montelukast sodium	①⑥⑦⑧	Remission period	RCT
Liu Z	2015	63	63	43.9 ± 12.1	43.3 ± 11.9	24 W	Modified Yupingfeng Powder+Salmeterol Xinafoate and Fluticasone Propionate Powder for Inhalation	Salmeterol Xinafoate and Fluticasone propionate powder for inhalation	①	Chronic persistent phase	RCT
Song X	2022	40	40	52.45 ± 2.59	52.68 ± 2.84	2 W	Yupingfeng Granules+Budesonide suspension for inhalation	Budesonide suspension for inhalation	①②③④⑥	Acute phase	RCT
Ma K	2020	39	39	38.5 ± 5.8	37.5 ± 5.7	12 W	Yupingfeng Granules+Budesonide and Formoterol Fumarate Powder for Inhalation	Budesonide and Formoterol Fumarate Powder for Inhalation	②③	Acute phase	RCT
Wang BJ	2019	120	120	48.53 ± 6.69	48.65 ± 6.79	2 W	Yupingfeng Dropping Pills+Fluticasone propionate inhaled aerosol	Fluticasone propionate inhaled aerosol	①②④	Chronic persistent phase	RCT
Wang K	2024	52	52	57.29 ± 10.06	58.86 ± 11.27	12 W	Yupingfeng Granules+Montelukast sodium	Montelukast sodium	①②③⑤	Remission period	RCT
Zhang D	2019	45	45	66.2 ± 11.2	65.8 ± 10.3	4 W	Yupingfeng Granules+Fluticasone propionate inhaled aerosol	Fluticasone propionate inhaled aerosol	①⑥⑦⑧	Not mentioned	RCT
Zhang J	2018	50	50	54.5 ± 5.2	54.3 ± 5.3	24 W	Yupingfeng Dropping Pills+Budesonide and Formoterol Fumarate Powder for Inhalation	Budesonide and formoterol fumarate powder for inhalation	①②⑤	Chronic persistent phase	RCT
Zhang JU	2022	38	38	40.63 ± 3.92	40.55 ± 3.87	12 W	Yupingfeng Granules+Budesonide and Formoterol Fumarate Powder for Inhalation	Budesonide and formoterol fumarate powder for inhalation	①	Chronic persistent phase	RCT
Zhong ZY	2016	60	60	38.5 ± 10.3	38.5 ± 10.3	2 W	Yupingfeng Granules+Montelukast sodium	Montelukast sodium	①②	Remission period	RCT
Zhou YS	2019	23	19	38.5 ± 12.4	38.5 ± 12.4	12 w	Yupingfeng Granules+Budesonide and Formoterol Fumarate Powder for Inhalation	Budesonide and Formoterol Fumarate Powder for Inhalation	⑨	Chronic persistent phase	RCT
Guo JL	2021	40	40	43.19 ± 5.68	43.279 ± 5.38	4 w	Modified Yupingfeng Powder+Budesonide and Formoterol Fumarate Powder for Inhalation	Budesonide and Formoterol Fumarate Powder for Inhalation	②③	Acute phase	RCT
Yang ZJ	2021	58	58	36.48 ± 5.62	35.27 ± 5.59	8 w	Yupingfeng Powder+Salmeterol Xinafoate and Fluticasone Propionate Aerosol	Salmeterol Xinafoate and Fluticasone Propionate Aerosol	⑥⑦⑧	Not mentioned	RCT
Li J	2022	48	48	48.28 ± 11.81	48.32 ± 11.74	4 w	Yupingfeng powder+budesonide suspension for inhalation	Budesonide suspension for inhalation	②⑤⑨	Remission period	RCT

① Overall clinical efficacy. ② FEV1. ③ FVC. ④ FEV1/FVC. ⑤ PEF⑥CD4+. ⑦ CD8+. ⑧ CD4+/CD8+. ⑨ACT scores. Abbreviations: T, treatment Group; C, control Group; W, week; RCT, Randomized Controlled Trial.

**TABLE 3:** Composition, Processing Technology, and quality control characteristics of YPF formulation in the included studies.

Author	Year of Publication	Type of Yupingfeng powder preparation	Core composition (Astragalus/Atractylodes/Saposhnikovia)	Processing technology	Quality control standards	Compliance with pharmacopoeia (Chinese Pharmacopoeia 2025 Edition)
Chen QL	2025	Granules	30 g Astragalus, 15 g Atractylodes, 10 g Saposhnikovia (crude drug equivalent/day)	Extracted by water decoction, spray-dried and granulated with particle size of 0.15∼0.5 mm	Granule size, solubility and water content conform to the general rules for Chinese medicinal granules; TLC identification of astragaloside Ⅳ and atractylenolide Ⅲ	Yes
Fang XY	2022	Granules	24 g Astragalus, 12 g Atractylodes, 8 g Saposhnikovia (crude drug equivalent/day)	Extracted by alcohol-water dual extraction, concentrated and dry-granulated with solid content ≥75%	Content determination: astragaloside Ⅳ ≥0.08%/g, saposhnikovia chromone glycoside ≥0.10%/g; microbial limit meets the specified requirements	Yes
Feng XH	2025	Powder (crude drug powder)	15 g Astragalus, 10 g Atractylodes, 6 g Saposhnikovia (crude drug powder/day)	Purified, crushed, sieved through 80-mesh sieve and mixed uniformly	Granule size and weight variation conform to the general rules for Chinese medicinal powders; microscopic identification of characteristic powder of the three medicinal materials	Yes
Fu ZL	2024	Granules	30 g Astragalus, 15 g Atractylodes, 10 g Saposhnikovia (crude drug equivalent/day)	Water extraction and alcohol precipitation, concentrated into clear paste for granulation with drying temperature of 60∼70 °C	Solubility and disintegration time limit meet the requirements; TLC identification of the three medicinal materials	Yes
Hou X	2021	Granules	20 g Astragalus, 10 g Atractylodes, 7 g Saposhnikovia (crude drug equivalent/day)	Decocted twice with water, the filtrates were combined and concentrated, then spray-dried for granulation	Water content ≤8.0%, qualified rate of granule size ≥95%; content determination of atractylenolide Ⅲ ≥0.05%/g	Yes
Huang ML	2023	Granules	25 g Astragalus, 12 g Atractylodes, 9 g Saposhnikovia (crude drug equivalent/day)	Alcohol extraction followed by water precipitation, concentrated and dried, granulated and sized	Weight variation within ±5%; TLC identification of characteristic components of Astragalus and Saposhnikovia	Yes
Li MJ	2020	Modified Decoction	30 g Astragalus, 15 g Atractylodes, 10 g Saposhnikovia + 6 g Pericarpium Citri Reticulatae, 10 g Poria cocos (core medicinal materials unchanged)	Traditional water decoction: 800 mL water added and decocted to 200 mL, taken warm twice a day	No unified quality standard, prepared in accordance with clinical decoction operation specifications; all medicinal materials are genuine products in the Pharmacopoeia	Core medicinal materials comply, no conflict with the Pharmacopoeia for modified medicinal materials
Liu Z	2015	Modified Decoction	25 g Astragalus, 12 g Atractylodes, 8 g Saposhnikovia +10 g Codonopsis pilosula, 6 g Radix Glycyrrhizae Preparata (core medicinal materials unchanged)	Traditional water decoction: 600 mL water added and decocted to 150 mL, 1 dose per day	No unified quality standard, decoction process in accordance with TCM clinical specifications; all medicinal materials are processed into genuine products in the Pharmacopoeia	Core medicinal materials comply, no conflict with the Pharmacopoeia for modified medicinal materials
Song X	2022	Granules	20 g Astragalus, 10 g Atractylodes, 7 g Saposhnikovia (crude drug equivalent/day)	Water extraction and concentration to make soft material, extruded for granulation, sieved after drying	Microbial limit: bacterial count ≤1000 cfu/g, mold and yeast count ≤100 cfu/g	Yes
Ma K	2020	Granules	24 g Astragalus, 12 g Atractylodes, 8 g Saposhnikovia (crude drug equivalent/day)	Spray-dried for granulation with particle fluidity ≥60s/100 g	Content determination of astragaloside Ⅳ ≥0.07%/g; complete dissolution in solubility test without precipitation	Yes
Wang BJ	2019	Dropping Pills	18 g Astragalus, 9 g Atractylodes, 6 g Saposhnikovia (crude drug equivalent/day)	Alcohol-water extraction and concentration, mixed with polyethylene glycol 6,000 at a ratio of 1:3, dropped and condensed	Weight variation within ±10%; disintegration time limit ≤30 min; TLC identification of the three medicinal materials	Yes
Wang K	2024	Granules	30 g Astragalus, 15 g Atractylodes, 10 g Saposhnikovia (crude drug equivalent/day)	Water extraction twice, the filtrates were combined and concentrated to a relative density of 1.20∼1.25 for granulation	Water content ≤7.0%, content determination of saposhnikovia chromone glycoside ≥0.09%/g	Yes
Zhang D	2019	Granules	25 g Astragalus, 12 g Atractylodes, 9 g Saposhnikovia (crude drug equivalent/day)	Dry granulation with raw materials of Chinese medicinal extract powder + microcrystalline cellulose (adjuvant ratio 1:0.2)	Particle size distribution: 0.2∼0.4 mm accounting for ≥90%; disintegration time limit ≤15 min	Yes
Zhang J	2018	Dropping Pills	20 g Astragalus, 10 g Atractylodes, 7 g Saposhnikovia (crude drug equivalent/day)	Water extraction and concentration, mixed with polyethylene glycol 4,000, dropping temperature of 80∼85 °C	Disintegration time limit ≤25 min; weight variation conforms to the general rules for Chinese medicinal dropping pills	Yes
Zhang JU	2022	Granules	24 g Astragalus, 12 g Atractylodes, 8 g Saposhnikovia (crude drug equivalent/day)	Water extraction and concentration, spray-dried into powder, granulated and packaged	TLC identification of characteristic components of Astragalus and Atractylodes; microbial limit meets the specified requirements	Yes
Zhong ZY	2016	Granules	20 g Astragalus, 10 g Atractylodes, 7 g Saposhnikovia (crude drug equivalent/day)	Traditional water decoction extraction, concentrated for granulation and dried to constant weight	Water content ≤9.0%, solubility conforms to the requirements for Chinese medicinal granules	Yes
Zhou YS	2019	Granules	25 g Astragalus, 12 g Atractylodes, 9 g Saposhnikovia (crude drug equivalent/day)	Alcohol-water dual extraction and concentration, granulated with particle size of 0.2∼0.6 mm	Content determination of atractylenolide Ⅲ ≥0.06%/g; weight variation meets the requirements	Yes
Guo JL	2021	Modified Decoction	30 g Astragalus, 15 g Atractylodes, 10 g Saposhnikovia + 6 g Pinellia ternata, 9 g Armeniacae Semen Amarum (core medicinal materials unchanged)	Traditional water decoction: 700 mL water added and decocted to 200mL, 1 dose per day	No unified quality standard, decoction in accordance with clinical specifications; all medicinal materials are genuine products in the Pharmacopoeia	Core medicinal materials comply, no conflict with the Pharmacopoeia for modified medicinal materials
Yang ZJ	2021	Powder (crude drug powder)	15 g Astragalus, 10 g Atractylodes, 6 g Saposhnikovia (crude drug powder/day)	Purified, cut, crushed at low temperature, sieved through 100-mesh sieve and mixed uniformly	Granule size and water content conform to the general rules for Chinese medicinal powders; microscopic identification of medicinal material characteristics	Yes
Li J	2022	Powder (decoction type)	20 g Astragalus, 10 g Atractylodes, 7 g Saposhnikovia (crude drug powder/day)	Crushed, sieved through 60-mesh sieve, decocted with water and taken 1 dose per day	All medicinal materials are genuine products in the Pharmacopoeia, and the crushing particle size meets the clinical decoction requirements	Yes

1. Raw material equivalent/day refers to the weight of the original raw medicinal materials corresponding to the daily dosage. All modified decoctions retain the three core components of YPF, with only adjuvant botanical ingredients added without altering the core formula. 2. Processing techniques follow conventional Chinese botanical preparation methods:powders are divided into raw powder and decoction-form powders; drop pills are all based on polyethylene glycol (PEG) as the matrix; granules primarily employ water extraction or dual extraction (water-alcohol) as the core extraction method.

### 3.3 Bias risk assessment

**FIGURE 2:**
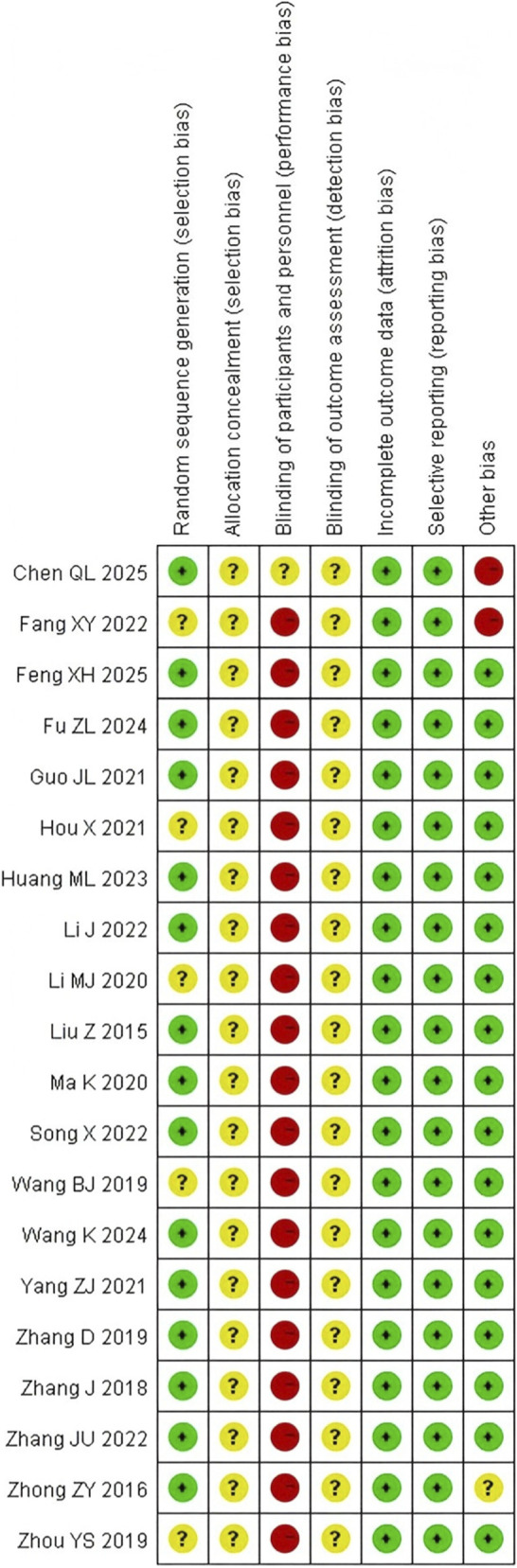
Risk of bias summary.

The overall methodological quality of the included studies was moderate. Fifteen studies (Zheng and Zhang, 2015; Zhong et al., 2016; Zhang, 2018; Zhang et al., 2019; Guo, 2021; Yang et al., 2021; Li et al., 2022; Ma, 2022; Song et al., 2022; Jun and Yu, 2022; Mulan et al., 2023; Fu and Dong, 2024; Wang and Wang, 2024; Chen, 2025; Xiuhua et al., 2025) specified random sequence generation methods, classified as low risk. All studies failed to clearly describe allocation concealment, classified as unclear risk. All studies were rated as “high risk” or “some concern” for “blinding of participants and investigators.” All outcome data were complete, with no bias due to missing data. After sequentially excluding studies without blinding or allocation concealment reporting, the clinical overall response rate was 1.18 (95% CI: 1.13–1.23), consistent with the original results, indicating that bias risk did not substantially affect core efficacy evaluation. Specific bias risk assessment results are shown in Figure 2.

### 3.4 Meta-analysis of clinical efficacy

**FIGURE 3:**
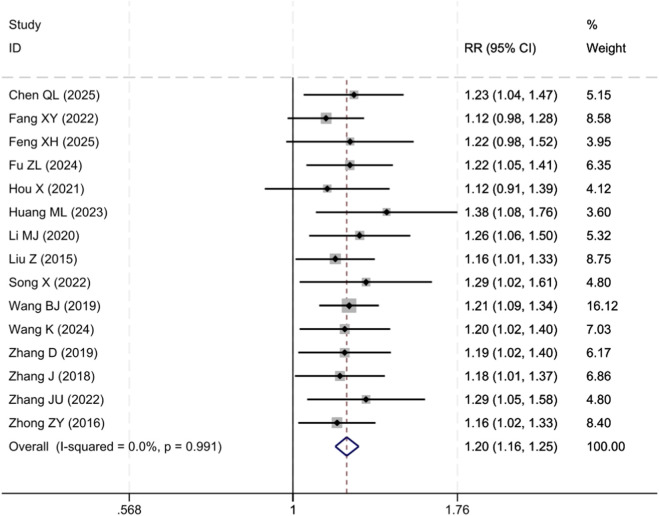
Forest plot comparing clinical efficacy between the YPF+CBT group and the CBT group.

Fifteen studies (Zheng and Zhang, 2015; Zhong et al., 2016; Zhang, 2018; Wang et al., 2019; Zhang et al., 2019; Minjie et al., 2020; Xin and Wenjuan, 2021; Fang and Dong, 2022; Song et al., 2022; Jun and Yu, 2022; Mulan et al., 2023; Fu and Dong, 2024; Wang and Wang, 2024; Chen, 2025; Xiuhua et al., 2025) evaluated the overall clinical response rate, with 742 participants each enrolled in the YPF+CBT group and the CBT group. The meta-analysis results demonstrated that the overall clinical response rate in the YPF+CBT group was significantly higher than that in the CBT group [RR = 1.20, 95% CI (1.16, 1.25), P < 0.001]. The interstudy heterogeneity was extremely low (I^2^ = 0.0%), and the results showed high consistency (Figure 3).

In this study, the overall clinical efficacy rate of YPF combined with CBT was 86.8%, while that of CBT alone was 72.3%. The absolute risk reduction (ARR) was 14.5%, and the number needed to treat (NNT) was 7, indicating that for every 7 adult asthma patients treated, 1 additional patient would achieve clinical efficacy. This suggests that the combined therapy offers clear clinical benefits.

### 3.5 Meta-analysis of pulmonary function parameters

#### 3.5.1 FEV1

**FIGURE 4:**
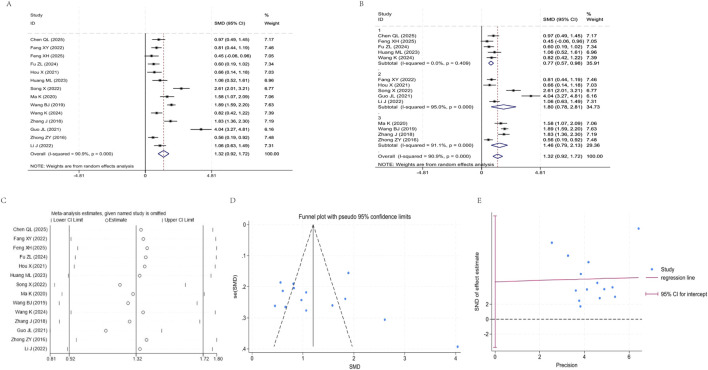
Meta-analysis of FEV1. **(A)** Forest plot. **(B)** Subgroup analysis. **(C)** Sensitivity analysis. **(D)** Funnel plot. **(E)** Egger plot.

Fifteen studies (Zhong et al., 2016; Zhang, 2018; Wang et al., 2019; Guo, 2021; Xin and Wenjuan, 2021; Fang and Dong, 2022; Li et al., 2022; Ma, 2022; Song et al., 2022; Mulan et al., 2023; Fu and Dong, 2024; Wang and Wang, 2024; Chen, 2025; Xiuhua et al., 2025) were included in the analysis, exhibiting high heterogeneity (I^2^ = 90.9%, P < 0.001). The pooled effect from the random effects model demonstrated that the YPF+CBT group showed significantly better improvement in FEV1 compared to the CBT group [SMD = 1.32, 95% CI (0.92, 1.72), P < 0.001]. After stratifying by study year into three subgroups, subgroup 1 (2023–2025) exhibited minimal heterogeneity (I^2^ = 0.0%, P = 0.409), with a pooled SMD of 0.77 (95% CI: 0.57, 0.98). Subgroups 2 (2020–2022) and 3 (2016–2019) still showed high heterogeneity (I^2^ values of 95.0% and 91.1%, respectively, both P < 0.001), with pooled SMD of 1.80 (95% CI: 0.78, 2.81) and 1.46 (95% CI: 0.79, 2.13), respectively. The 95% CI for each subgroup did not cross the invalidation line. The funnel plot showed an asymmetric distribution, and Egger regression suggested potential publication bias. Sensitivity analysis revealed a narrow fluctuation range for the pooled SMD (approximately 0.81–1.80), indicating good stability of the results (Figure 4).

#### 3.5.2 FVC

**FIGURE 5:**
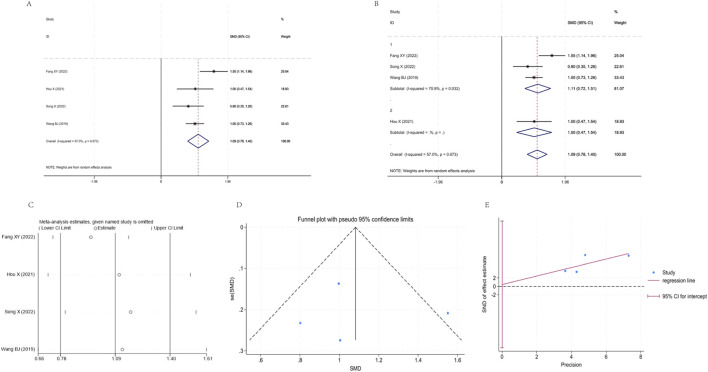
Meta-analysis of FVC. **(A)** Forest plot. **(B)** Subgroup analysis. **(C)** Sensitivity analysis. **(D)** Funnel plot. **(E)** Egger plot.

Seven studies (Guo, 2021; Fang and Dong, 2022; Ma, 2022; Song et al., 2022; Mulan et al., 2023; Wang and Wang, 2024; Chen, 2025) were included in the analysis, exhibiting high interstudy heterogeneity (I^2^ = 95.2%, P < 0.001). The pooled random effects model demonstrated that the YPF+CBT group showed significantly better FVC improvement than the CBT group [SMD = 1.78, 95% CI (0.91, 2.64), P < 0.001]. After stratification by treatment duration into two subgroups, subgroup 1 still exhibited high heterogeneity (I^2^ = 96.8%, P < 0.001), with a pooled SMD of 2.66 (95% CI: 1.06, 4.25); subgroup 2 showed resolved heterogeneity (I^2^ = 0%, P = 0.883) and a pooled SMD of 0.70 (95% CI: 0.44, 0.96). Both groups indicated significant improvement with combination therapy. Sensitivity analysis revealed minimal variation in effect sizes (0.63–3.02), demonstrating good result stability. Funnel plot combined with Egger regression suggested potential publication bias (Figure 5).

#### 3.5.3 FEV1/FVC

**FIGURE 6:**
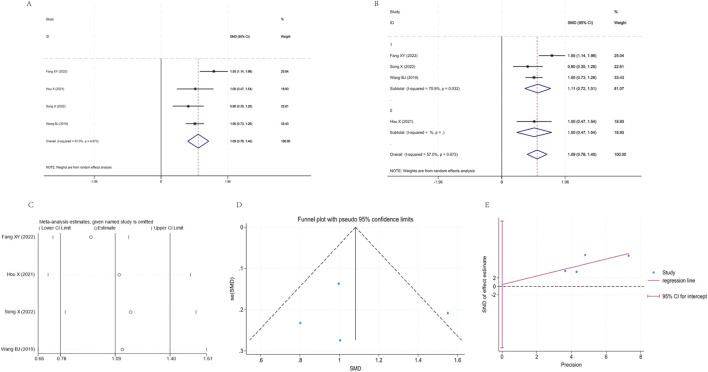
Meta-analysis of FEV1/FVC. **(A)** Forest plot. **(B)** Subgroup analysis. **(C)** Sensitivity analysis. **(D)** Funnel plot. **(E)** Egger plot.

Four studies (Wang et al., 2019; Xin and Wenjuan, 2021; Fang and Dong, 2022; Song et al., 2022) were included in the analysis, with moderate heterogeneity among the studies (I^2^ = 57.0%, P = 0.073). Analysis of the random effects model showed that the YPF+CBT group demonstrated significantly better improvement in the FEV1/FVC ratio compared to the CBT group [SMD = 1.09, 95% CI (0.78, 1.40), P < 0.05]. Subgroup analysis suggested that individual studies were potential sources of heterogeneity but did not alter the core conclusions. Sensitivity analysis confirmed the robustness of the results. Funnel plot combined with Egger’s test indicated no significant publication bias (Figure 6).

#### 3.5.4 PEF

**FIGURE 7:**
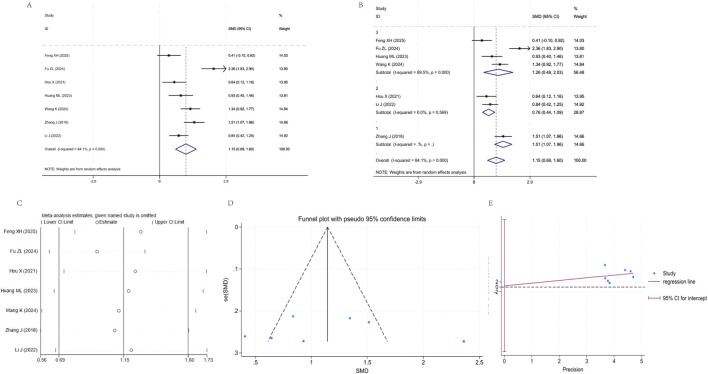
Meta-analysis of PEF. **(A)** Forest plot. **(B)** Subgroup analysis. **(C)** Sensitivity analysis. **(D)** Funnel plot. **(E)** Egger plot.

Seven Studies (Zhang, 2018; Xin and Wenjuan, 2021; Li et al., 2022; Mulan et al., 2023; Fu and Dong, 2024; Wang and Wang, 2024; Xiuhua et al., 2025) were included in the analysis, with high heterogeneity among studies (I^2^ = 84.1%, P = 0.000). The pooled random effects model demonstrated that the PEF improvement effect in the YPF+CBT group was significantly superior to that in the CBT group [SMD = 1.15, 95% CI (0.69, 1.60), P < 0.05]. Subgroup analysis divided the studies into three subgroups, with subgroup 2 showing 0% heterogeneity (P = 0.569), suggesting it as a potential source of heterogeneity. However, the pooled effects of all subgroups and the overall analysis were positive. Sensitivity analysis revealed that the effect sizes with 95% CI overlapped with the original results after sequentially excluding individual studies, indicating good conclusion stability. Due to the limited number of studies, the funnel plot’s symmetry interpretation was restricted, and the Egger test suggested no significant publication bias (Figure 7).

### 3.6 Meta-analysis of other outcomes

#### 3.6.1 CD4+/CD8+ ratio

**FIGURE 8:**
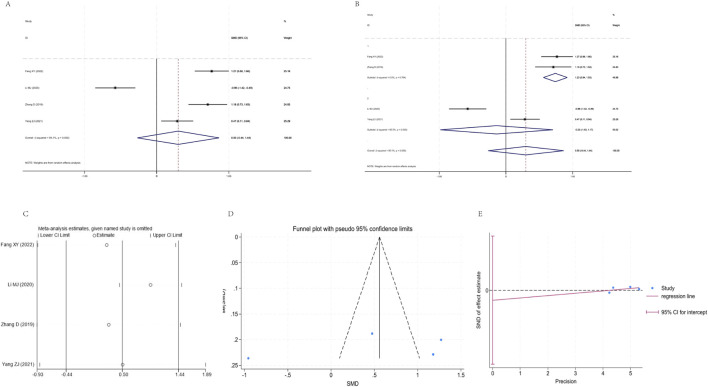
Meta-analysis of CD4+/CD8+. **(A)** Forest plot. **(B)** Subgroup analysis. **(C)** Sensitivity analysis. **(D)** Funnel plot. **(E)** Egger plot.

Four studies (Zhang et al., 2019; Minjie et al., 2020; Yang et al., 2021; Fang and Dong, 2022) were included in the analysis, exhibiting extremely high heterogeneity (I^2^ = 95.1%, P = 0.000). The pooled random effects model showed no statistically significant difference in the CD4+/CD8+ratio between the YPF+CBT group and the CBT group [SMD = 0.50, 95% CI (−0.44, 1.44), P = 0.301]. Subgroup analysis revealed 0% heterogeneity in subgroup 1(P = 0.764), where YPF significantly improved the CD4+/CD8+ ratio [SMD = 1.23, 95% CI (0.94, 1.53)]. Subgroup 2 still showed high heterogeneity (95.5%), and the pooled results were not statistically significant [SMD = −0.23, 95% CI (−1.63, 1.17)]. Sensitivity analysis indicated considerable variability in effect sizes, suggesting poor conclusion stability. Funnel plot combined with Egger’s test indicated a potential risk of publication bias (Figure 8).

#### 3.6.2 ACT scores

**FIGURE 9:**
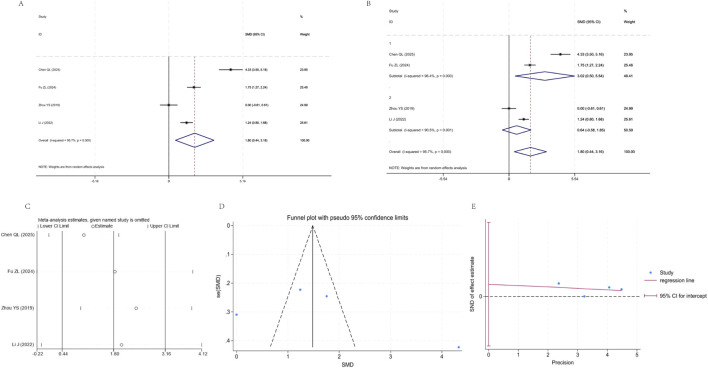
Meta-analysis of ACT. **(A)** Forest plot. **(B)** Subgroup analysis. **(C)** Sensitivity analysis. **(D)** Funnel plot. **(E)** Egger plot.

Four studies (Zhou et al., 2019; Li et al., 2022; Fu and Dong, 2024; Chen, 2025) were included in the analysis, exhibiting high heterogeneity (I^2^ = 95.7%). The pooled random-effects model demonstrated that the YPF+CBT group had significantly higher ACT scores than the CBT group [SMD = 1.80, 95% CI (0.44, 3.16), P = 0.010]. Subgroup analysis revealed significant effects in some subgroups but no statistical significance in others, with heterogeneity attributed to study characteristics. Sensitivity analysis indicated good conclusion stability. Funnel plot combined with Egger’s test suggested a certain risk of publication bias (Figure 9).

### 3.7 Analysis of heterogeneity sources

#### 3.7.1 Subgroup analysis

To investigate the consistency of efficacy between YPF+CBT and CBT and potential sources of heterogeneity, subgroup analyses were conducted based on treatment duration, asthma stage, study year, sample size, and botanical type. All subgroups confirmed that YPF+CBT was significantly superior to CBT in improving clinical efficacy: the pooled RR for the subgroup with treatment duration>8 weeks was 1.221 (95% CI: 1.143, 1.305; P = 0.000); the pooled RR for the small sample size subgroup (n < 40) was 1.246 (95% CI: 1.135, 1.368; P = 0.000); the pooled RR for the granule subgroup was 1.206 (95% CI: 1.143, 1.273; P = 0.000); the subgroup with treatment duration 4–8 weeks (RR = 1.167, 95% CI: 1.026, 1.326; P = 0.019) and the subgroup with modified decoction (RR = 1.195, 95% CI: 1.074, 1.330; P = 0.001) also showed statistical significance; A single study investigating the decoction formulation yielded an RR of 1.217 (95% CI: 0.978, 1.516; P = 0.079), which suggested a positive therapeutic trend but no statistically significant difference; this null finding may be attributed to the limited sample size of the individual study, while the overall efficacy trend was consistent with that of other subgroups. The I^2^ value in all subgroups was 0.0% (all P-values for heterogeneity testing>0.5), indicating no significant heterogeneity within subgroups (Table 4).

**TABLE 4:** Subgroup analysis based on clinical efficacy.

5. Herb type						
Granule	10	449/449	1.206	[1.143, 1.273]	0	0.00%
Powder	1	30/30	1.217	[0.978, 1.516]	0.079	-
Modified powder	2	103/103	1.195	[1.074, 1.330]	0.001	0.00%
Dropping pill	2	170/170	1.201	[1.103, 1.309]	0	0.00%

#### 3.7.2 Meta-regression analysis

To further quantify the sources of heterogeneity, a multivariate meta-regression analysis was conducted using a random effects model based on 15 RCTs reporting clinical efficacy. Five potential confounding variables, including treatment duration and asthma stage, were included. The model fit was good (F = 0.30, P = 0.9024), with residual heterogeneity I^2^_res = 0.00%, indicating complete explanation of interstudy heterogeneity. The results showed that treatment duration (P = 0.03, coefficient = −0.0034) and study year group (P = 0.043, coefficient = −0.0191) were significant sources of heterogeneity, while asthma stage, sample size, and botanical type had no significant impact on heterogeneity (all P > 0.05). These findings were highly consistent with subgroup analysis results (Table 5).

**TABLE 5:** Meta-regression analysis results.

Variable	Coefficient	Std. err.	t-value	P>|t|	95% confidence interval
Treatment duration	−0.0034038	0.0287744	−0.12	0.03	[−0.0684959, 0.021683]
Asthma stage	0.0137214	0.024037	0.57	0.582	[−0.0406542, 0.068097]
Sample size	−0.0238046	0.0477744	−0.5	0.63	[−0.131878, 0.0842687]
Study year group	−0.0190956	0.0389833	−0.49	0.043	[−0.107282, 0.0390907]
Herb type	0.0241104	0.0418828	0.58	0.579	[−0.0706351, 0.1188558]
Constant (_cons)	1.209618	0.1043572	11.59	0	[0.9735461, 1.445691]

Meta-regression model parameters:number of observations = 15; REML estimate of between-study varianceτ^2^ = 0; residual heterogeneity I-squared_res = 0.00%; adjusted R-squared for variance explained between studies = .%; F (5, 9) for all covariates in the joint test model = 0.30; corrected Prob> F = 0.9024(Knapp-Hartung correction).

### 3.8 Safety analysis

Among the 20 studies included in this review, only two explicitly reported adverse reactions, both of which were mild. These included gastrointestinal discomfort (abdominal distension, nausea) and dry mouth. No statistically significant difference in incidence was observed between the intervention group and the control group (P > 0.05).

## 4 Discussion

In the past 5 years, research on TCM botanical formulations for asthma treatment has achieved significant progress, with accomplishments in TCM botanical formulations compound formulations, monomers, and external TCM botanical formulations therapies. For instance, compound studies have demonstrated that Xiaoqinglong Decoction (Wang T et al., 2025) and Yanghepingchuan Decoction (Yuan D. et al., 2025) combined with CBT can improve pulmonary function and reduce inflammatory cytokines. Monotherapy studies have confirmed the protective effects of quercetin and Perilla seed oil on asthma mice (Fang et al., 2023; Xie, 2008). External TCM botanical formulations therapies represent a viable option for asthma patients intolerant to oral medications (Xu et al., 2013). Despite these advances, high-quality evidence for YPF—a classical Chinese herbal formula—in adult bronchial asthma remains scarce, and no systematic review or meta-analysis focusing on YPF combined with CBT for adult bronchial asthma has been identified. This study, as the first systematic review and meta-analysis specifically addressing this topic, aims to evaluate the therapeutic effects of YPF and provide reliable references for clinical application.

### 4.1 Clinical efficacy and safety study

This study demonstrated that YPF+CBT exhibited significant clinical benefits in the treatment of adult bronchial asthma: ① The overall clinical response rate was 14.5% higher than that of CBT alone; ② Significant advantages were observed in pulmonary function-related parameters (FEV1, FVC, FEV1/FVC ratio, PEF) and ACT scores.

In terms of safety, YPF+CBT demonstrated good short-term tolerability, with only mild adverse reactions such as gastrointestinal discomfort and dry mouth observed in two of the 20 included studies; no serious adverse events were reported in either group, and the incidence rates showed no statistically significant difference compared to CBT alone (P > 0.05). However, since only 10% of studies reported safety outcomes and there is a lack of unified criteria for adverse reaction assessment, these limitations hinder comprehensive evaluation of the potential risks associated with long-term YPF application and introduce uncertainty for its clinical promotion.

### 4.2 Heterogeneity analysis

This study found that the clinical overall response rate exhibited extremely low statistical heterogeneity (I^2^ = 0.0%), whereas pulmonary function parameters showed high heterogeneity (FEV1 I^2^ = 90.9%, FVC I^2^ = 95.2%, PEF I^2^ = 84.1%). The ACT scores and CD4+/CD8+ ratio also demonstrated high heterogeneity (I^2^ values were 95.7% and 95.1%, respectively). To identify the sources of heterogeneity, subgroup analyses were conducted based on treatment duration, asthma stage, study year, sample size, and YPF formulation type, with meta-regression used for validation. Results indicated that all subgroups supported the superiority of YPF+CBT over CBT, with heterogeneity eliminated within subgroups (I^2^ = 0.0%). Meta-regression further confirmed that treatment duration (P = 0.03) and study year group (P = 0.043) were significant sources of heterogeneity, while asthma stage, sample size, and YPF formulation type had no significant impact (all P > 0.05). Notably, the CD4+/CD8+ ratio showed no statistically significant difference (SMD = 0.50, 95% CI (−0.44, 1.44), P = 0.301), which may be attributed to the small sample size and significant baseline immune status variations among patients. This suggests that the immunomodulatory effects of YPF may only manifest in specific populations and require further validation.

### 4.3 Methodological quality and limitations of the study

The RCTs included in this study exhibited moderate overall methodological quality, with the following issues: None of the studies explicitly described allocation concealment methods, and effective blinding was not implemented for participants and investigators, resulting in a high risk of performance bias—a common deficiency in clinical research on TCM botanical formulations. Due to the distinct morphological and olfactory characteristics of YPF granules and decoctions, achieving double-blind placebo simulation proved challenging. However, sensitivity analysis confirmed that after excluding studies without blinding measures, the pooled RR for overall clinical efficacy (1.18, 95% CI: 1.13–1.23) remained highly consistent with the original result (1.20, 95% CI: 1.16–1.25), indicating that bias did not substantially affect core conclusions and that the therapeutic advantage of combination therapy was reliable.

Furthermore, small sample sizes are a major limitation of individual RCTs, with most studies having fewer than 60 participants, which can lead to insufficient statistical power and increased randomization errors. This study combined 20 RCTs through meta-analysis, achieving a total sample size of 1,896 cases. Subgroup analyses stratified by sample size demonstrated that statistical power exceeded the minimum clinical research threshold of 80% across all subgroups (n < 40, 40–60, and n > 60), with all efficacy outcomes showing statistical significance. These findings confirm that the therapeutic advantage of YPF remains unaffected by the sample size of individual studies.

In addition to the aforementioned methodological issues, this study also has other limitations: all included studies were published in China, resulting in geographical and linguistic biases, and conclusions should be cautiously extrapolated to other ethnic and regional populations; diagnostic criteria for TCM syndrome types are not unified, with 30% of studies failing to clearly label syndrome type information, making it difficult to precisely define the optimal population for YPF; some outcome indicators were included in a limited number of studies, resulting in limited statistical power and incomplete clarification of heterogeneity sources. These limitations point out directions for improvement in future research.

## 5 Conclusion

In conclusion, YPF combined with CBT can significantly improve clinical efficacy, pulmonary function, and symptom control in adult patients with bronchial asthma. However, due to limitations such as methodological quality of included studies, geographical constraints, and insufficient adverse event data, these findings require further validation through high-quality RCTs.

## Data Availability

The original contributions presented in the study are included in the article/Supplementary Material, further inquiries can be directed to the corresponding authors.
