# Supplementary material for: Efficacy of Yupingfeng powder in the treatment of bronchial asthma in adults: a systematic review and meta‐analysis
Source: Front Pharmacol. 2026 May 1;17:1757474. doi: 10.3389/fphar.2026.1757474 (PMC13176258; doi:10.3389/fphar.2026.1757474)
Supplement: Supplementary file 1 [file Table1.docx]

**Efficacy of Yupingfeng Powder in the Treatment of Bronchial Asthma in Adults: A Systematic Review and Meta-analysis**

**Supplement: content**

| **TABLE S1a. Search strategy in Pubmed** ([https://pubmed. ncbi. nlm. nih. gov/](https://pubmed.ncbi.nlm.nih.gov/)) | |
| --- | --- |
| Asthma | 1. Asthma[MeSH Terms]  2. Asthmas[MeSH Terms]  3. Bronchial Asthma[MeSH Terms]  4. Asthma bronchiale[Title/Abstract]  5. Asthma pulmonale[Title/Abstract]  6. Asthmatic[MeSH Terms]  7. Asthmatic subject[MeSH Terms]  8. Bronchus asthma[MeSH Terms]  9. Chronic asthma[MeSH Terms]  10. Lung allergyObstructions[MeSH Terms]  11. 1 OR 2 OR 3 OR 4 OR 5 OR 6 OR 7 OR 8 OR 9 OR 10 |
| **Yuping feng san** | 12.Yupingfen[MeSH Terms]  13.YPFS herbal formulation[MeSH Terms]  14. Yu ping feng dai pao cha[MeSH Terms]  15. Yu ping feng san[MeSH Terms]  16.Yu ping feng jiao nang[MeSH Terms]  17.Yu ping feng ke li[MeSH Terms]  18.Yu ping feng kou fu ye[MeSH Terms]  19.12 OR 13 OR 14 OR 15 OR 16 OR 17 OR 18 |
| **Study Type** | 20. "Clinical Trial" [Publication Type]  21. "Randomized Controlled Trial" [Publication Type]  22. 20 OR 21 |
| **Combine** | 23. 11 AND 19 AND 22 |

| **TABLE S1b. Search strategy in EMBASE (**<https://www.embase.com/>**)** | |
| --- | --- |
| **Asthma** | #1 'asthma'/exp  #2 'asthma':ti,ab,kw  #3 'asthmas':ti,ab,kw  #4 'bronchial asthma':ti,ab,kw  #5 'asthma bronchiale':ti,ab,kw  #6 'asthma pulmonale':ti,ab,kw  #7 'asthmatic':ti,ab,kw  #8 'asthmatic subject':ti,ab,kw  #9 'bronchus asthma':ti,ab,kw  #10'chronic asthma':ti,ab,kw  #11 'lung allergy':ti,ab,kw  #12 #1 OR #2 OR #3 OR #4 OR #5 OR #6 OR #7 OR #8 OR #9 OR #10 OR #11 |
| **Yuping feng san** | #13 'yupingfeng'/exp  #14 'ypfs herbal formulation':ti,ab,kw  #15 'yu ping feng':ti,ab,kw  #16 'yu ping feng dai pao cha':ti,ab,kw  #17 'yu ping feng jiao nang':ti,ab,kw  #18 'yu ping feng ke li':ti,ab,kw  #19 'yu ping feng kou fu ye':ti,ab,kw  #20 'yu ping feng san':ti,ab,kw  #21 13 OR 14 OR 15 OR 16 OR 17 OR 18 OR 19 OR 20 |
| **Study Type** | #22 'controlled clinical trial'/exp  #23 'randomized controlled trial'/exp  #24 #22 OR #23 |
| **Combine** | #12 AND #21 AND #24 |

| **TABLE S1c. Search strategy in Web Of Science(**<https://www.webofscience.com/>**)** | |
| --- | --- |
| **Asthma** | 1. TS=(asthma)  2. TS=(Asthmas)  3. TS=(Bronchial Asthma)  4. TS=(asthma bronchiale)  5. TS=(asthma pulmonale)  6. TS=(asthmatic)  7. TS=(asthmatic subject)  8. TS=(bronchus asthma)  9. TS = (chronic asthma)  10. TS = (lung allergy)  11.1 OR 2 OR 3 OR 4 OR 5 OR 6 OR 7 OR 8 OR 9 OR 10 |
| **Yuping feng san** | TS = (Yupingfeng)  TS = (YPFS herbal formulation)  TS = (yu ping feng)  TS = (yu ping feng dai pao cha)  TS = (yu ping feng jiao nang)  TS = (yu ping feng ke li)  TS = (yu ping feng kou fu ye)  TS = (yu ping feng san)  12 OR 13 OR 14 OR 15 OR 16 OR 17 OR 18 OR 19 |
| **Study Type** | 21. TS=(Clinical Trial)  22. TS=(Randomized Controlled Trial)  23. 21 OR 22 |
| **Combine** | 24. 11 AND 20 AND 23 |

| **TABLE S1d. Search strategy in Cochrane(**<https://www.cochrane.org/>**)** | |
| --- | --- |
| **Asthma** | #1 MeSH descriptor: [asthma] explode all trees |
| **Yuping feng san** | #2 (Yupingfeng):ti,ab,kw  #3 (YPFS herbal formulation):ti,ab,kw  #4 #2 OR #3 |
| **Study Type** | #5 ("clinical trial"):ti,ab,kw  #6 ("randomized controlled trial"):ti,ab,kw  #7 (RCT):ti,ab,kw  #8 #5 OR #6 OR #7 |
| **Combine** | #9 #1 AND #4 AND #8 |

| **TABLE S1e. Search strategy in Commonly Used Databases for Research in China** | |
| --- | --- |
| **CNKI (**<https://www.cnki.net/>**)** | (SU%= 'Yupingfeng' OR SU%= 'Yiqi Gubiao') AND (SU%= 'asthma' OR SU%= 'xiao zheng' OR SU%= 'asthma') |
| **WanFangdata(**<https://www.wanfangdata.com.cn/>**)** | Subject: (Yupingfeng or Yiqi Gubiao) and Subject: (asthma or xiao zheng or asthma) |
| **Sinomed**(<http://www.sinomed.ac.cn>) | ("Yupingfeng" [Common Field: Smart] OR "Yiqi Gubiao" [Common Field: Smart]) AND ("Asthma" [Common Field: Smart] OR "Xiao Zheng" [Common Field: Smart] OR "asthma" [Common Field: Smart]) |
